# [Corrigendum] S100A8-targeting siRNA enhances arsenic trioxide-induced myeloid leukemia cell death by down-regulating autophagy

**DOI:** 10.3892/ijmm.2026.6034

**Published:** 2026-11-02

**Authors:** Liangchun Yang, Minghua Yang, Hong Zhang, Zhuo Wang, Yan Yu, Min Xie, Mingyi Zhao, Liying Liu, Lizhi Cao

Int J Mol Med 29: 65-72, 2012; DOI: 10.3892/ijmm.2011.806

Following the publication of the above article, it was drawn to the Editor's attention by a concerned reader that, for the western blots shown in Fig. 4C, the two left lanes in the 'Fibrillarin' gel slice appeared to be strikingly similar to the mirrored two right lanes in the 'Actin' gel slice, albeit the orientations of the blots were horizontally reversed, such that data which were intended to have shown the results of differently performed experiments appeared to have been derived from the same original source. Moreover, the control β-actin blots featured in Fig. 5A of the above paper were strikingly similar to control blots used in a different context that appeared in a subsequently published paper featuring some of the same authors in *American Journal of Cancer Research*.

Following the publication of an expression of concern statement (doi: 10.3892/ijmm.2025.5622) that drew attention to these issues, we have now received a response to these anomalies from the authors. They explained that the re-use of the control β-actin blots featured in Fig. 5A in the paper featuring some of the same authors in *American Journal of Cancer Research* had occurred in error, although these data were presented correctly in the above article; the authors would contact the Editor of *American Journal of Cancer Research* in order to request a corrigendum for the other article. Regarding the duplication of the western blots in Fig. 4C, the blots correctly shown for the fibrillarin gel slice had inadverently been copied across for the actin blots. However, the authors were able to consult their original images (also presenting the raw data for the blots to the Editorial Office), and prepared a revised version of Fig. 4, which now features the correct data for the actin blots in Fig. 4C. The revised version of Fig. 4 is shown on the next page. The authors confirm that the error made during the assembly of this figure did not have a significant impact on either the results or the conclusions reported in this study, and all the authors agree with the publication of this Corrigendum. The authors are grateful to the Editor of *International Journal of Molecular Medicine* for allowing them the opportunity to publish this Corrigendum; furthermore, they apologize to the readership of the Journal for any inconvenience caused.

## Figures and Tables

**Figure 4.**
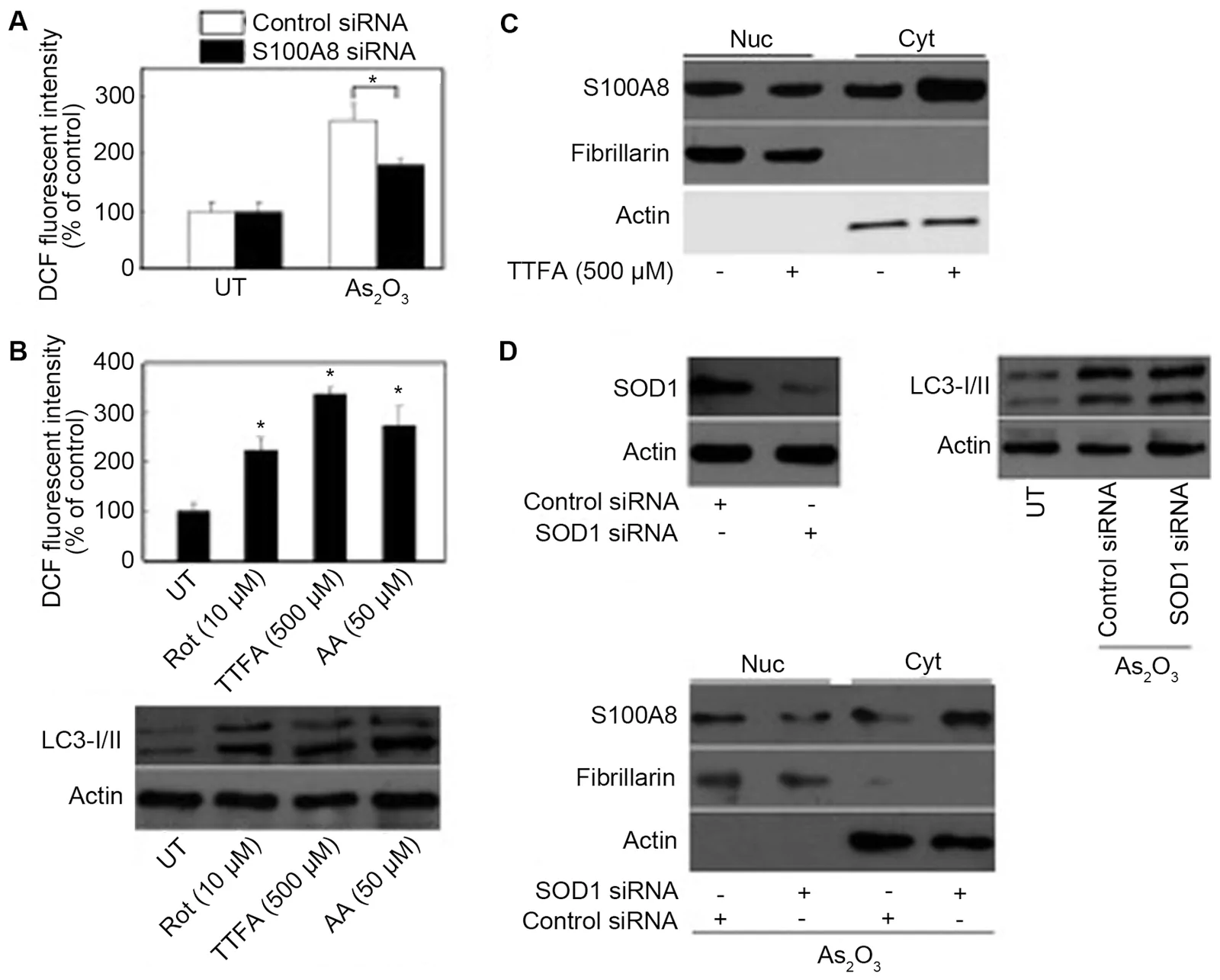
Reactive oxygen species (ROS)-dependent signals enhance autophagy and regulate the S100A8 translocation. (A) Knockdown of S100A8 limits As2O3-induced ROS production. HL-60 cells were transfected with S100A8 siRNA for 48 h, and then treated with As2O3 for 24 h. ROS production was assessed by measuring the fluorescence intensity of DCF in a fluorescence plate reader. The increasing production of ROS was expressed as a percentage of control. (n=3, ^*^P<0.05). UT, untreated group. (B) mECT inhibitors promote ROS production and autophagy. HL-60 cells were stimulated with rotenone (Rot), thenoyltrifluoroacetone (TTFA), and antimycin A (AA) at the indicated doses for 12 h. ROS production was assessed by measuring the fluorescence intensity of DCF in a fluorescence plate reader and LC3-Ⅰ/Ⅱ expression level was assayed by Western blot analysis. The increasing production of reactive oxygen species was expressed as a percentage of control. (n=3, ^*^P<0.05 vs. the untreated group). UT, untreated group. (C) mECT inhibitors promote S100A8 translocation HL-60 cells were treated with TTFA (500 *μ*M) for 24 h, and the nuclear/cytosolic S100A8 expression was assayed by Western blot analysis. Fibrillarin is a nuclear fraction control, and actin is a cytoplasmic fraction control. Nuc, nuclear; Cyt, cytosolic. (D) SOD1 RNAi limits As2O3-induced autophagy and S100A8 translocation. HL-60 cells were pre-treated with SOD1 siRNA for 48 h. Then cells were treated with As2O3 for 24 h, the nuclear/cytosolic S100A8 expression and LC3-Ⅰ/Ⅱ expression were assayed by Western blot analysis. Nuc, nuclear; Cyt, cytosolic; UT, untreated group.

